# Continuous Automated Model EvaluatiOn (CAMEO)—Perspectives on the future of fully automated evaluation of structure prediction methods

**DOI:** 10.1002/prot.26213

**Published:** 2021-08-19

**Authors:** Xavier Robin, Juergen Haas, Rafal Gumienny, Anna Smolinski, Gerardo Tauriello, Torsten Schwede

**Affiliations:** ^1^ Biozentrum University of Basel Basel Switzerland; ^2^ Computational Structural Biology SIB Swiss Institute of Bioinformatics Basel Switzerland

**Keywords:** benchmarking, blind assessment, continuous evaluation, macromolecular complexes, non‐canonical residues, molecular structure prediction, ligands

## Abstract

The Continuous Automated Model EvaluatiOn (CAMEO) platform complements the biennial CASP experiment by conducting fully automated blind evaluations of three‐dimensional protein prediction servers based on the weekly prerelease of sequences of those structures, which are going to be published in the upcoming release of the Protein Data Bank. While in CASP14, significant success was observed in predicting the structures of individual protein chains with high accuracy, significant challenges remain in correctly predicting the structures of complexes. By implementing fully automated evaluation of predictions for protein–protein complexes, as well as for proteins in complex with ligands, peptides, nucleic acids, or proteins containing noncanonical amino acid residues, CAMEO will assist new developments in those challenging areas of active research.

## 1 INTRODUCTION

The 2020 CASP14 experiment saw an unprecedented improvement in the performance of three‐dimensional (3D) protein structure prediction. One method (AlphaFold2) was able to generate highly accurate predictions even for the most challenging de novo targets. Beyond the CASP community, this breakthrough has implications for the entire field of structural biology: accurately predicting the structure of a single protein chain has never been closer to being considered a solved problem. But far from being the end of structure prediction, this might instead be the beginning of a new era in the 3D modeling of biomolecular structures. Areas that have been limited so far due to the inability to produce sufficiently accurate de novo protein models in the first place, such as the prediction of protein–ligand interactions, large macromolecular complexes and assemblies, or variant effects, might now be within reach of the next generation of structural prediction methods. Independent blind assessment of these techniques will be more than ever required in order to support the development of reliable and reproducible methods. In order to assist the community to tackle those challenges, we are introducing an extension of Continuous Automated Model EvaluatiOn (CAMEO; available at https://beta.cameo3d.org) with the aim to shift the focus from the prediction of individual protein chains to the prediction of macromolecular complexes as determined experimentally by X‐ray crystallography or increasingly cryo‐EM techniques and deposited to the Protein Data Bank (PDB).^1^

In this new CAMEO category, participating methods receive the sequences of all unique polymer chains, as well as the InChI codes of nonpolymer entities composing the complex as prediction targets. The challenges of the modeling task are to: (1) predict the stoichiometry of the complex; (2) predict the 3D structure of all the components: proteins, peptides, DNA, RNA and ligands, including their orientation and interfaces; and (3) provide per‐residue confidence estimates of the model. This CAMEO category is based on an opt‐in model: participants only receive the target type(s) their method is able to model. This means that a method that only predicts single protein chains can still participate and will receive the targets composed of only one protein sequence, which can be either monomers or homo‐oligomers, while another method by the same group might be designed to predict, for example, complexes of proteins with drug‐like small molecules.

In this article, we describe the different types of prediction targets that CAMEO enables in the new category, and estimate the number of expected validation targets for each category based on PDB statistics observed in 2020. One major challenge will be the scoring of the new type of predictions with regard to the actual experimental structures. Wherever appropriate, we comment on scores that are foreseen to be applied to the various prediction types. We are welcoming feedback from the community regarding complementary scoring approaches.

## 2 MATERIAL AND METHODS

### 2.1 Sequence filtering and clustering

The prerelease sequences of polymer entities as well as InChI code of nonpolymer ligands were downloaded every Saturday from the PDB^1^ (http://www.wwpdb.org/files/). Structures containing sequences with unknown residues, starting with caps, or whose type (protein, DNA, or RNA) could not be assigned unambiguously were discarded. Within a prerelease week, amino acid sequences of 30 amino acid residues or longer (“protein”) were clustered with CD‐HIT^2^ applying a 99% sequence identity threshold. Amino acid sequences of less than 30 amino acid residues (“peptides”), as well as DNA and RNA sequences were clustered based on exact identity (100%). One representative sequence per cluster was selected as target for structure prediction.

### 2.2 Template searches

Target protein sequences were submitted to two template searches. First, a BLAST+ v. 2.2.31^3^ search against a database of current PDB entries at the time of prerelease was performed. A threshold of 85% sequence identity and at least 70% coverage was used to identify target sequences with very high similarity to a protein with known structure. Next, sequence profiles were built using 1 iteration of HHblits v. 3.2.0^4^ against Uniclust30 (2018_08).^5^ The profiles were used to search a database of PDB entries available on March 19, 2021, with an HHblits probability threshold of 70% and a coverage threshold of 70% in order to identify target sequences with more remote similarity to a protein with a known structure. Since this was done as a retrospective analysis, hits that were released after the date of the prerelease of the target were filtered out. For peptide sequences of less than 30 amino acid residues and sequences of nucleic acid residues, a lookup was performed against a database of current PDB entries at the time of prerelease with a 100% identity threshold.

Templates found by BLAST, HHblits and lookup on single chains were aggregated into complexes. A structure was considered to be a template if all the chains of the target structure could be uniquely mapped to the chains of the template structure, and the template structure did not contain any extra polymer chain.

### 2.3 Scores

Single‐chain predictions were evaluated against the reference structure with the lDDT score^6^ using OpenStructure v. 2.1.0,^7^ the global CAD atom–atom (AA) score v. 1646_63d6b800098c,^8^ and the GDT_TS score using LGA v. 05/2009.^9^ Model confidence assessed the ability of predictors to estimate the quality of their own models, as described elsewhere.^10^ When the target structure contained more than one copy of the sequence, more than one biological assembly, or for homo‐oligomeric predictions, the scores were calculated between all possible combinations of target assembly and target and model chains, and only the most favorable score was kept.

Homo‐ and hetero‐oligomeric predictions were evaluated with the oligo‐lDDT and QS‐score^11^ using OpenStructure v. 2.1.0,^7^ as well as the MM‐align‐based TM‐score v. 20 190 426.^12^ The oligomeric lDDT score (oligo‐lDDT) is an extension of the lDDT score for protein complexes and has also been used in CASP since CASP13.^13^, ^14^ It relies on the QS‐score to identify the mapping of chains and residues between the model and target structure. Once the mapping is identified, the all‐atom lDDT score can be applied on the protein complex in the same way as it is applied for single chains with the advantage that it now also considers inter‐chain contacts. Extra atoms in the model for mapped chains have no effect on lDDT scores, while extra atoms in the target structure reduce the score. For the oligomeric lDDT score, we penalize extra chains in both reference and model by including them as nonconserved contacts.

### 2.4 Target difficulty

Based on the “model‐1” prediction results of all public servers, targets were classified as “hard” if the average lDDT was smaller than 0.5, “easy” if the average lDDT was 0.75 or higher, and “medium” otherwise, as described elsewhere.^10^

### 2.5 Quality estimation

The first models (model‐1) from public servers were harvested approximately 24–30 h after the submission of sequences to 3D servers. ROC AUC, partial ROC AUC (0.0–0.2 FPR), PR AUC, and partial PR AUC (0.8–1.0 TPR) were calculated with an lDDT threshold of 0.6, as described elsewhere.^10^, ^15^

### 2.6 Ligand analysis

Functional domain annotation was extracted from CATH^16^ version 4.3.0. We used the “Structure external” links from DrugBank^17^ version 5.1.8 to identify drug‐containing targets. The analysis was performed with Python 3.6.6, OpenStructure v. 2.1.0,^7^ and pandas v. 1.1.5.^18^

### 2.7 Structure visualization

Structural figures were generated with the Mol* Viewer.^19^

## 3 RESULTS AND DISCUSSIONS

### 3.1 Current CAMEO results

Since 2012, CAMEO has been leveraging the prerelease of structures to be published in the upcoming release of the PDB to conduct weekly, blind, fully automated benchmarking experiments. Every Saturday, we download the prerelease data, which contains the sequences of polymer entities, as well as InChI codes of nonpolymer entities contained in the PDB structures to be released on the following Wednesday. We select a set of 20 interesting protein‐modeling targets that are submitted to registered participants, who have 4 days to predict the 3D structure of those targets. We collect those predictions and, upon release of the structures by the PDB on Wednesdays, compare the predictions with the experimental ground truth.

The CAMEO evaluation provides a wide variety of scores measuring different aspects of protein structure prediction accuracy, and accordingly does not establish a single unique ranking between the methods. However, some of the scores are featured more prominently on the web site, as we consider them more useful estimations of the model quality. The focus of CAMEO has always been on all‐atom scores to capture the ability of participants to accurately model proteins including biologically relevant protein side chain conformations. In addition, as CAMEO is a fully automated workflow without human intervention, we have been focusing on superposition‐free scores which alleviate the need to manually split proteins into evaluation units^20^, ^21^ to account for domain movements. Therefore, CAMEO has been showcasing scores like lDDT^6^ and CAD‐score,^8^ both of which are all atom scores and superposition independent. In addition, our server summary page features the lDDT‐BS score which measures the accuracy of predictions in the region of ligand binding sites, as well as a measure for model confidence, which evaluates the ability of participants to estimate the accuracy of their own predictions. Additional scores such as GDT,^9^ RMSD, and TM‐score^12^, ^22^ are displayed on the target details page and available in the downloads; however, they are not aggregated as the results are misleading due to the nature of superposition based scores and their inherent limitation when applied to multi‐domain proteins.

Since 2016, CAMEO^10^ has been evaluating the ability of modeling servers to correctly predict the oligomeric state of a target protein and model the correct assembly, based solely on the amino acid sequence. As targets are submitted as a single protein sequence, participants need to predict whether the protein is likely to assemble into a homo‐oligomer and, if that is the case, to predict the exact stoichiometry as well as the correct interfaces. The complex models are evaluated with the oligo‐lDDT score,^15^ which is a modified version of lDDT that looks at the whole complex and accounts for missing or extra chains; the MM‐align‐based^12^ TM‐score and RMSD, which are superposition‐dependent; and the QS‐score,^11^ which looks specifically at the conservation of interface residues.

In 2020, we performed 52 prediction rounds and provided targets to 15 public modeling servers (from nine groups) and 25 development servers (from a total of 18 groups). After filtering problematic targets of low or uncertain quality, or targets causing technical issues to scoring tools for formatting reasons, we evaluated and scored 812 targets, 453 of which were oligomeric. Table 1 shows a summary of the target structures that were released by the PDB, the experimental method, as well as the clustering and selection status, for all targets as well as those that were scored as homo‐oligomers. Results of the public servers are shown in Table S1. Compared with 84 3D modeling targets of CASP14, CAMEO enables participants to assess the accuracy of their prediction servers on a wide variety of targets in much shorter time intervals.

**TABLE 1:** Number of targets of each experimental type released by the PDB in 2020, remaining after clustering, and selected for submission

		Released by the PDB					Clustering	Selection
		Total	X‐ray	EM	Solution NMR	Other		
Current CAMEO		15 028	12 551	2182	247	48	7466	1038
	of which homo‐oligomeric	4494	3823	631	20	20	2341	405
Protein complexes	all	12 901	10 570	2050	235	46	7511	4141
	only proteins	11 566	9705	1604	212	45	6465	3158
	of which hetero‐oligomers	2304	1361	930	11	2	1496	1130
	… homo‐oligomers	4032	3383	608	20	21	2284	1011
	… monomers	5230	4961	66	181	22	2685	1017
Protein–ligand complexes	all	9929	8577	1298	31	23	8889	3567^a^
	only protein‐small molecule	9040	8007	979	31	23	8094	3491^a^
	of which hetero‐oligomers	1543	939	598	6	0	1235	296^a^
	… homo‐oligomers	3218	2873	335	1	9	2904	1291^a^
	… monomers	4278	4195	45	24	14	3954	1904^a^
Peptide complexes	all	749	614	56	68	11	605	536
	only peptides	107	40	5	51	11	90	83
	of which hetero‐oligomers	6	6	0	0	0	6	5
	… homo‐oligomers	23	16	5	1	1	23	22
	… monomers	78	18	0	50	10	61	56
DNA complexes	all	513	280	208	25	0	391	390
	only DNA	61	33	4	24	0	58	57
	of which hetero‐oligomers	13	6	4	3	0	12	12
	… homo‐oligomers	28	24	0	4	0	26	25
	… monomers	20	3	0	17	0	20	20
RNA complexes	all	422	123	275	21	3	327	323
	only RNA	78	48	12	16	2	45	42
	of which hetero‐oligomers	14	10	0	4	0	6	6
	… homo‐oligomers	8	8	0	0	0	6	4
	… monomers	56	30	12	12	2	33	32
Mixed complexes		1335	865	446	23	1	1046	983
	protein‐peptide	608	563	28	17	0	483	421
	protein‐RNA	243	46	191	5	1	200	199
	protein‐DNA	381	225	155	1	0	279	279
	protein‐RNA–DNA	69	20	49	0	0	52	52
	protein‐RNA‐peptide	32	9	23	0	0	30	30
	protein‐RNA‐peptide	2	2	0	0	0	2	2
Complexes with noncanonical residues		1075	940	113	20	2	666	444
	proteins	824	717	103	3	1	496	286
	peptides	198	180	0	17	1	124	112
	RNA	34	22	12	0	0	28	27
	DNA	52	52	0	0	0	35	35

^a^ For protein–ligand complexes, the selection criterion includes both the existence of closely related homolog complexes in the PDB and the presence of the ligands in DrugBank.

### 3.2 Quality estimation

Every Sunday, approximately 24–30 h into the evaluation cycle, we collect models that have been already returned by public 3D participating servers. We submitted these models as prediction targets for quality estimation. Throughout 2020, we, hence, collected 8594 models. Results of the evaluation of public servers are shown in Table S2.

### 3.3 Protein complexes

The new version of CAMEO extends the scope of the assessment to structures and complexes. Instead of considering every protein sequence separately, a prediction target is now defined as a complete experimental structure with all the chemical entities it contains. In the case of monomeric and homo‐oligomeric protein entries, this would be identical to the current CAMEO‐3D targets and contain only one unique protein sequence. However, for hetero‐oligomeric targets, evaluation is only performed in the context of the whole complex, and no longer as individual isolated protein chains taken out of context. Methods registered to receive hetero‐oligomeric complexes as targets thus receive all sequences of the proteins that form a complex, and are expected to predict the oligomeric structure of the complex. All participating methods receive the sequences of monomeric or homo‐oligomeric targets. This allows establishing a common baseline where all participating servers can be compared with each other on a subset of common targets.

**FIGURE 1:**
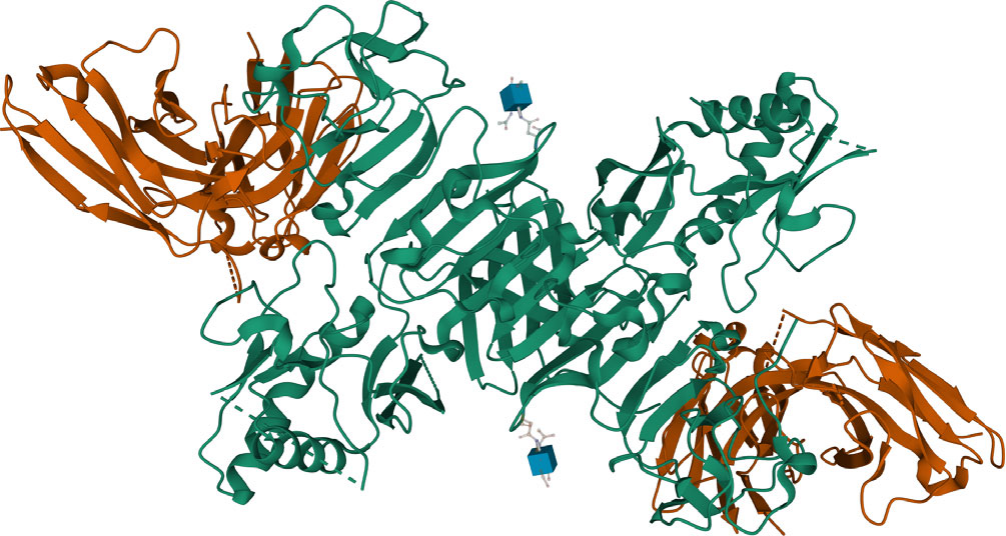
Target 2020‐12‐19_00000231 (PDB ID 7 K93) is a hetero‐2‐2‐mer protein complex of a Dengue virus nonstructural protein (NS1) (green) in complex with a mouse neutralizing single chain Fab variable region (orange).^23^ While templates can be easily identified with HHblits for both entities, there is no overlap between the template lists, meaning the two proteins have never been observed in a homologous complex. Specifically, no homologs of this Dengue virus protein have been observed in complex with an antibody. Hence, this constitutes an interesting target for modeling heteromeric protein complexes

In order to select interesting targets for this category, we search for the presence of homologous complexes (Figure 1). Closely related homologs are first identified with BLAST for every protein sequence with 30 or more amino acid residues separately. Complexes containing DNA, RNA, or peptide sequences shorter than 30 amino acids are excluded at this stage, and handled separately (see following sections). For every target, we consider the complete set of proteins that compose it, and search for a homologous template that covers all the protein entities. We ignore templates that only cover some of the target sequences, or that contain extra polymer entities (proteins, peptides, DNA, or RNA). We consider targets to be interesting if such a closely related homologous complex cannot be found. This includes cases of novel complexes (where all the proteins can be modeled separately easily, but where the complex has never been observed experimentally in its entirety, and therefore the interface(s) is unknown) or if at least one of the protein sequences in the complex is a nontrivial modeling target on its own.

Looking at the data we collected in the 52 prerelease weeks of 2020, 3158 interesting protein structures where no closely related homolog could be found with BLAST were released by the PDB. Among those, 1017 were monomers, 1011 homo‐oligomeric complexes (which cannot be distinguished from monomers from the sequence‐only prerelease data) and 1130 were hetero‐oligomers (Table 1).

In order to retrospectively analyze the complexity of the hetero‐oligomeric target set, we repeated the template search with HHblits to identify more remotely related homologous complexes. We could identify a homologous hetero‐oligomeric complex with HHblits for 565 of these 1130 targets, where all entities of the target could be uniquely mapped to the template, and reciprocally. In 240 hetero‐oligomeric complexes, templates for individual entities could be identified with HHblits, but not in the same complex (or the template contained extra entities); and 113 complexes could similarly be identified with BLAST. These 353 “novel complex” targets are of particular interest, as an accurate prediction would have to successfully predict the assembly mode of the complex, and accurately model the (unknown) interfaces, therefore going beyond the classical reach of homology modeling. Finally for the remaining 212 complexes, no template could be identified by HHblits for at least one of the target entities.

HHblits was able to identify homologs in the vast majority (1734) of the 2028 monomeric (1017) or homo‐oligomeric (1011) interesting protein structures contained in the CAMEO target set. We note, however, that 43 of the targets could only be mapped to templates in complex with a different partner. The interfaces are likely to differ from the templates, and therefore we consider these targets as interesting modeling targets for CAMEO. Finally, HHblits was unable to identify a template for 294 of these targets.

**FIGURE 2:**
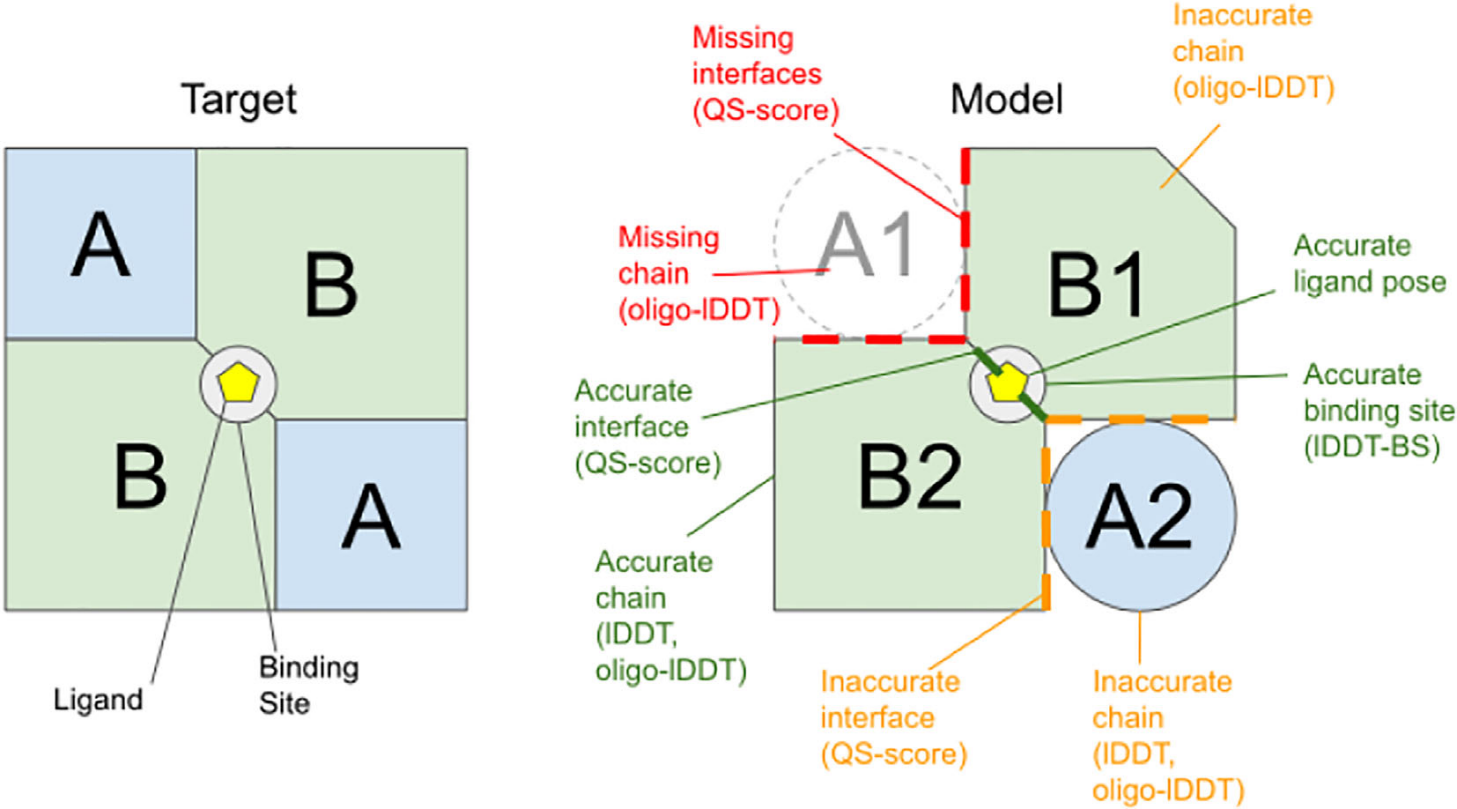
Hypothetical hetero‐2‐2‐mer target (AABB, left) with a ligand, and a hypothetical model of the target (right). (1) The lDDT score assesses the accuracy of each individual chain and measures local and global differences between model and reference structure. When more than one chain is predicted for an entity (B1, B2), only the best‐scoring one (B2) is kept. (2) The oligo‐lDDT score assesses the accuracy of all chains simultaneously while penalizing for missing (A1) or extra chains. (3) The QS‐score assesses the accuracy of the interface(s) between chains. It identifies correct (green dashed line) and inaccurate (orange dashed line) interfaces, and penalizes missing (red dashed line) interfaces. (4) The lDDT‐BS score assesses the accuracy of the binding site of biologically relevant ligands (gray circle, center). (5) Ligand scores assess the accuracy of the ligand (yellow) pose

In order to evaluate the predictions, we are using the same scores as for the homo‐oligomers: oligo‐lDDT, QS‐score, and TM‐score. In addition, other single‐chain scores can be generalized to evaluate heteromers in the same fashion as the oligo‐lDDT score is a generalization of the lDDT score to oligomers. Figure 2 shows the main scores for this category, and highlights the different aspects of modeling they are assessing. Finally, we are also looking at the applicability of the scores used by the CAPRI community for automated evaluation.

It should be noted that the selection of interesting protein target structures is performed regardless of ligand contents, but nonpolymer ligands are submitted nonetheless to participating servers that support it. Seventy‐six percent of the structures released by the PDB in 2020, and 65% of the interesting protein structures selected in this category, contain at least one ligand. In addition, we are considering specifically selecting interesting ligand modeling targets, which we describe in the following section.

### 3.4 Protein–ligand complexes

Small chemical compounds, which are not part of a polymer chain, are provided as InChI codes and PDB chemical components in the prerelease of the PDB. They are included in the target definition together with the polymer entities for participating servers that support predicting small chemical compounds in complex with proteins. Consequently, in addition to predicting the correct protein structure, predictors are challenged to include the ligands in their models at the correct binding site in an accurate conformation.

However, predicting the exact pose of a ligand within a theoretical model remains a challenge which is out of reach for most current protein prediction servers. To specifically facilitate the development of such methods, these should be evaluated separately to the prediction of protein complexes. Therefore, we are proposing a specialized CAMEO category, where easy protein modeling targets (as per the opposite of the definition in the previous section) are selected if they contain novel ligands that have not been seen in a template.

We analyzed the feasibility of this approach on the current data in the PDB. In 2020, we observed 4755 protein targets that would be trivial to solve with comparative modeling but included a combination of nonpolymer ligands never seen before in a template for those structures. Furthermore, 4398 of them contained only homo‐oligomeric or monomeric targets, which would enable many current protein structure prediction servers to participate without having to implement new modeling approaches for protein complexes.

**FIGURE 3:**
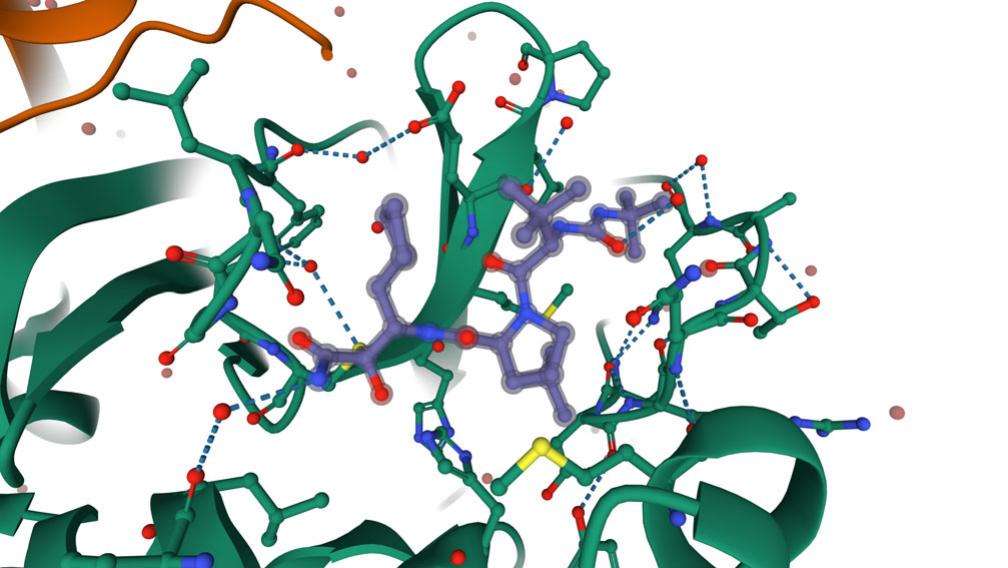
Target 2020‐05‐09_00000305 (PDB ID 7BRP) is a structure of the SARS‐CoV‐2 main protease in complex with Boceprevir.^24^ At the time of prerelease, the structure of the protease had already been solved, and was therefore a trivial modeling target on its own. However, it had not been observed in complex with Boceprevir, and therefore, this complex represents a challenging ligand modeling target

Interestingly, 3491 of these 4755 structures contained a known drug from DrugBank^17^ (Table 1). Figure 3 shows a typical example of such a target, the SARS‐CoV‐2 main protease in complex with Boceprevir, an FDA‐approved drug for the treatment of the hepatitis C virus.^24^ Drug repurposing studies are common in the PDB, and the CAMEO target set is therefore representative of current areas of active research and can help developers to assess the performance of their methods on relevant datasets. For instance, 149 DrugBank drug‐containing ligand‐modeling targets were identified by CATH as containing the 3CL‐PRO main protease domain 3 (CATH ID 1.10.1840.10), and an additional 70 targets had ligands not known to DrugBank.

To score these predictions, we will first follow the procedure developed by other ligand benchmarking efforts such as CELPP^25^ and the D3R Grand Challenges,^26^, ^27^, ^28^, ^29^ which evaluate ligand poses with a symmetry‐corrected RMSD. This metric is easy to compute and understand in the context of a ligand; however, it may overestimate the dynamics of solvent accessible groups. Other metrics will be investigated such as the distance RMSD (dRMSD), as well as measures of native ligand‐protein contacts, which are also being considered in CELPP, and would complement contact‐based scores frequently used for the scoring of protein models.

### 3.5 Peptides

Accurately predicting the structures of short proteins or peptides has always been challenging for comparative modeling. As a consequence, many protein prediction servers have limits on the minimal length of protein sequences that they attempt to predict. CAMEO has so far taken a conservative approach and submitted targets containing at least 30 amino acids to the participants. In the future, participants will be able opt‐in to also receive peptides with less than 30 residues as targets. These targets are relevant in several areas of research such as host‐pathogen interactions.

In order to identify interesting novel targets, we considered a conservative cutoff of 100% sequence identity to a template. In 2020, the PDB released 536 novel structures containing at least one amino acid sequence of less than 30 residues. In 453 structures, such peptides were in complex with a protein or DNA/RNA, making those structures suitable for instance for peptide‐protein docking methods. Eighty‐three structures contained only peptides, either in monomeric, homo‐oligomeric, or hetero‐oligomeric forms, mainly with NMR (Table 1). Advances in AI and de novo modeling technologies may very well make it feasible to predict the structure of those peptides.

The interface (QS‐score) and complex (oligo‐lDDT) scores can be used to score protein‐peptide complexes. However, additional scores like those used in the CAPRI experiment,^30^ DockQ^31^ and other scores geared toward protein–peptide docking, will also be considered.

### 3.6 DNA and RNA

Although several standalone approaches have been developed,^32^, ^33^ and fully automated web prediction servers^34^, ^35^, ^36^, ^37^ are available, predicting the 3D structure of nucleic acids, RNA in particular, remains a challenge and an area of active development.^38^, ^39^

Considering a conservative cutoff of 100% sequence identity with previously known structures to identify interesting novel targets, 323 new structures containing RNA were released by the PDB in 2020, and 390 containing DNA. In most of these structures, nucleic acids were in complex with proteins. Just 42 contained only RNA, and 57 only DNA (Table 1). This low number of modeling targets might prove a challenge for blind benchmarking of nucleic acid structure prediction methods.

Regarding the scoring, many of the scores applicable to protein models can be readily applied to nucleic acids too, and were reviewed in,^39^ in particular, the CAD‐score^40^ which is already used for proteins in CAMEO. Other all atom, superposition‐free scores will be considered too. In addition, more specialized scores that take the base‐pairing nature of RNA structures into account, such as the interaction network fidelity (INF) and deformation index (DI), will be considered.^39^

### 3.7 Mixed complexes

**FIGURE 4:**
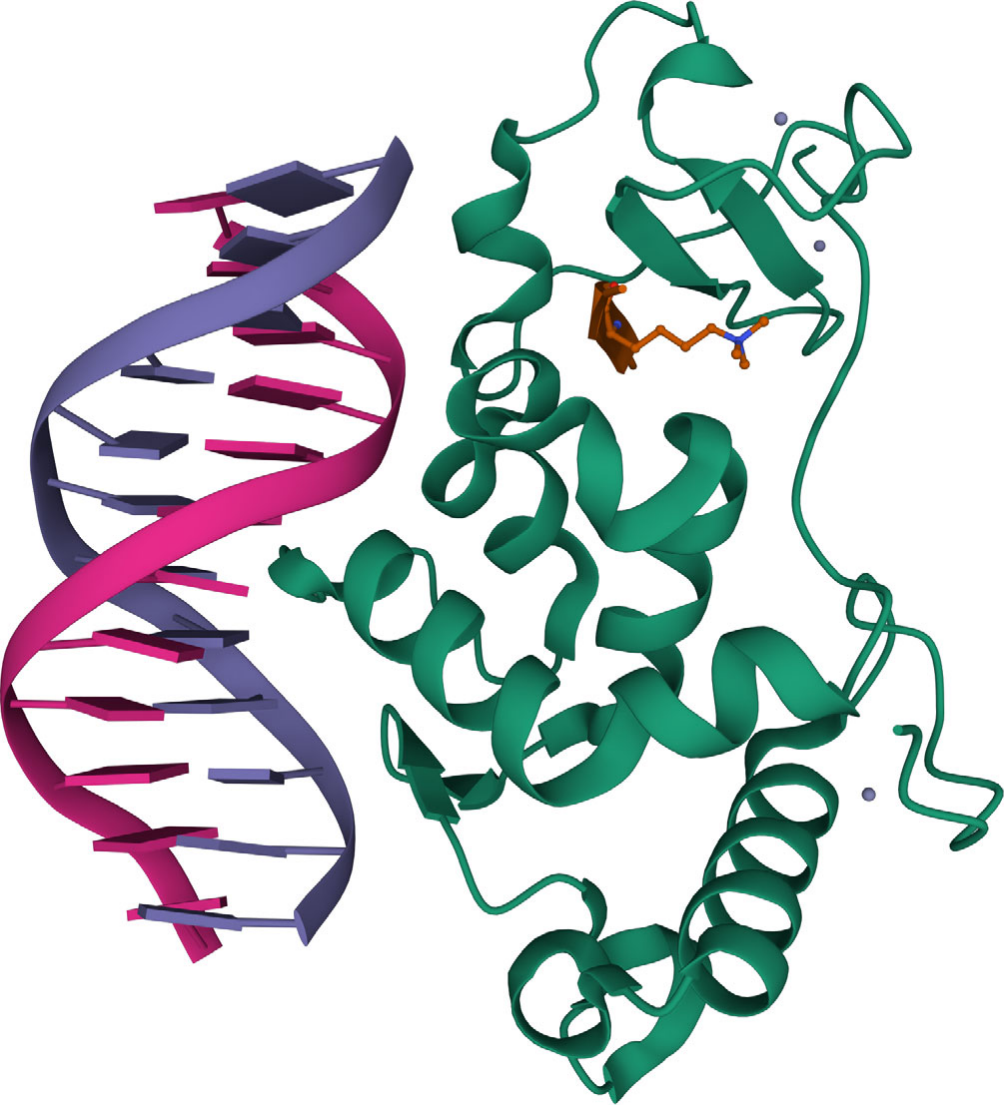
Target 2020‐05‐30_00000276 (PDB ID 6LQF) is an ARID‐PHD protein cassette in complex with a peptide, DNA, and zinc ions.^41^ The protein only has remote similarity (<30% sequence identity) to known structures, and none of them are in complex with DNA or the H3K4me3 peptide, making it an extremely challenging target. We are not aware of any methods that would currently be able to model this type of complex with acceptable accuracy. It should be noted that the peptide contains a noncanonical residue (*N*‐Trimethyllysine, derived from lysine)

Finally, CAMEO can submit targets containing a combination of all of the above: complexes with proteins, peptides, nucleic acids and ligands (Figure 4), thereby assessing the ability to predict any biologically relevant macromolecular structure, regardless of its composition. While this prediction task is extremely challenging for most methods to date, we believe this to be the ultimate goal in 3D structure prediction.

In 2020, following the criteria outlined in the previous sections, we observed 983 structures containing more than one type of polymer entities (Table 1). All of them were proteins in complex with peptides (421), DNA (279), RNA (199), DNA and RNA (52), or both peptides and nucleic acids (32).

With appropriate extensions, we believe that some of the scores selected for the individual target types such as the oligo‐lDDT and CAD‐score will be applicable to evaluate all these targets in a consistent manner.

### 3.8 Noncanonical amino acids and bases

Macromolecular structures frequently contain amino (or nucleic) acid residues which are not part of the 20 (respectively, 8) standard residues. Traditionally for modeling purposes, the target sequences are canonicalized, that is modified residues are represented by their “parent” or closest canonical amino acid residue. However, this may result in suboptimal models which would not accurately represent the region containing the modification. Posttranslational modifications such as phosphorylations can result in significant conformational changes of the protein structure, which would be impossible to correctly model without knowledge of the modification.

As this information is available at the time of prerelease, CAMEO can provide sequences containing noncanonical residues on an opt‐in basis (Figure 4). In this case, sequences will contain the PDB component identifier (typically three letters) enclosed in round brackets, in place of the parent amino acids. Models correctly representing those residues are expected to obtain higher scores for the all‐atom measures such as the lDDT or the CAD‐score.

In 2020, 444 of the 4323 protein, DNA, RNA, and mixed structures and complexes that we observed contained noncanonical residues (Table 1). We observed these noncanonical residues in proteins (286), peptides (112), DNA (35), and RNA (27). Sixteen of them were observed in mixed complexes.

### 3.9 Current implementation status of CAMEO

At the time of writing, the CAMEO “Structures & Complexes” functionality is available as a beta version at https://beta.cameo3d.org/ and is open for registrations. It has been providing targets containing proteins, DNA and RNA to registered servers on a weekly basis since October 2020. Participants can currently choose to receive the nonpolymer ligands contained in these targets as InChI codes or PDB component IDs, as well as noncanonicalized sequences including modified residues. Predictions can be returned in PDB or mmCIF format, and are assessed with a fully automated pipeline including the oligo‐lDDT and QS‐scores. A weekly download of models, reference structures, and assessment results are made available for offline analysis.

Our next steps will be to refine the target selection process, especially with respect to selecting relevant ligand targets as described in the previous sections. We are exploring ways to increase the diversity of the target selection, while ensuring that as many participants as possible receive a common subset of targets in order to make comparisons between servers possible for some aspects of the evaluation. We aim to improve the scoring by providing more diverse scores as described in the previous sections. Most groups developing novel methods have implemented their own scoring workflows locally. We therefore consider at this point the raw data downloads of the prediction results as a crucial service to the community developing specialized prediction methods as it allows including independent blind prediction data in publications describing the new method.

## 4 CONCLUSION

With the extension of CAMEO to the fully automated assessment of prediction of complexes (including protein–protein, DNA, RNA, peptides, and small molecules), we aim to encourage and facilitate the development of automated structure prediction servers going beyond the modeling of single chains of amino acids. In this article, we identified several challenging aspects of modeling which we believe will become more active areas of research in the future, and that are suitable for benchmarking with CAMEO. By assessing prediction targets with the same complexity as experimental structures using an “opt in” mechanism for the diverse modeling tasks, CAMEO will assist the development of new methods tackling these specific modeling challenges. As demonstrated by analyzing the PDB releases of the last year, CAMEO will be able to provide a diverse set of challenging blind prediction targets to enable the community to tackle next generation modeling challenges.

We welcome feedback from the community on which of these aspects should be prioritized and how various predictions should be numerically evaluated in CAMEO. We encourage methods developers to register to the beta CAMEO server to help testing and evolving these new features according to the needs of the prediction community.

### PEER REVIEW

The peer review history for this article is available at https://publons.com/publon/10.1002/prot.26213.

## Supporting information


**Table S1** 3D servers comparison for “hard” (182), “medium” (427), “easy” (203) and oligomeric (453) targetsᵃ in the 2020 time frame.

**Table S2** QE servers comparison for all targets (8594)ᵃ in the 2020 time frame.

## Data Availability

The data that support the findings of this study are available from the corresponding author upon reasonable request.
