# Supplementary material for: Continuous Automated Model EvaluatiOn (CAMEO)—Perspectives on the future of fully automated evaluation of structure prediction methods
Source: Proteins. 2021 Aug 19;89(12):1977–86. doi: 10.1002/prot.26213 (PMC8673552; doi:10.1002/prot.26213)
Supplement: Supplementary file 1 — Table S1 3D servers comparison for “hard” (182), “medium” (427), “easy” (203) and oligomeric (453) targetsᵃ in the 2020 time frame. [file PROT-89-1977-s002.pdf]

Supplementary Table 1: 3D servers comparison for "hard" (182), "medium" (427), "easy" (203) and oligomeric (453) targets[a] in the 2020 time frame.

Server name	Reference	Response time (hh:mm) - all	Returned fraction - hard	lDDT - hard	CAD score - hard	Returned fraction - medium	lDDT - medium	CAD score - medium	Returned fraction - easy	lDDT - easy	CAD score - easy	Model confidence - all	lDDT-BS - all	Returned fraction - oligo	QS-score - oligo	oligo-lDDT - oligo
tFold	[1]	48:00	0.92	0.56 (0.11)	0.61 (0.07)	0.95	0.70 (0.08)	0.69 (0.05)	0.94	0.82 (0.05)	0.76 (0.04)	0.56 (0.09)	0.68 (0.20)	0	-	-
BestSingleStructuralTemplate[c]	[2]	03:58	0.95	0.51 (0.11)	0.55 (0.08)	0.97	0.72 (0.08)	0.70 (0.06)	0.97	0.86 (0.05)	0.81 (0.04)	0.67 (0.13)	0.71 (0.24)	0	-	-
Robetta	[3–5]	23:50	0.98	0.49 (0.13)	0.56 (0.08)	0.98	0.71 (0.08)	0.69 (0.06)	1	0.83 (0.05)	0.78 (0.03)	0.83 (0.11)	0.68 (0.20)	0.57	0.52 (0.33)	0.69 (0.12)
IntFOLD6-TS	[6]	33:19	0.78	0.46 (0.11)	0.52 (0.08)	0.92	0.70 (0.08)	0.68 (0.06)	0.89	0.83 (0.04)	0.78 (0.03)	0.83 (0.10)	0.73 (0.23)	0	-	-
RaptorX	[7]	17:37	0.69	0.45 (0.12)	0.54 (0.07)	0.77	0.68 (0.09)	0.68 (0.05)	0.81	0.82 (0.04)	0.77 (0.03)	0.64 (0.08)	0.70 (0.19)	0	-	-
IntFOLD5-TS[b]	[8]	44:49	0.87	0.43 (0.11)	0.51 (0.08)	0.91	0.69 (0.09)	0.68 (0.06)	0.9	0.83 (0.04)	0.78 (0.03)	0.84 (0.11)	0.74 (0.23)	0	-	-
IntFOLD4-TS[b]	[9]	57:34	0.64	0.42 (0.12)	0.50 (0.08)	0.59	0.69 (0.09)	0.67 (0.06)	0.63	0.82 (0.04)	0.77 (0.03)	0.83 (0.12)	0.72 (0.25)	0	-	-
IntFOLD3-TS[b]	[10]	34:54	0.95	0.39 (0.13)	0.49 (0.08)	0.94	0.68 (0.09)	0.66 (0.06)	0.92	0.82 (0.04)	0.76 (0.04)	0.86 (0.10)	0.70 (0.24)	0	-	-
M4T-SMOTIF-TF[b]	[11]	09:42	0.29	0.38 (0.14)	0.42 (0.13)	0.85	0.66 (0.13)	0.64 (0.11)	0.99	0.82 (0.08)	0.76 (0.07)	0.71 (0.10)	0.71 (0.25)	0	-	-
SPARKS-X	[12]	06:31	0.78	0.34 (0.11)	0.46 (0.08)	0.85	0.58 (0.13)	0.60 (0.08)	0.78	0.74 (0.13)	0.71 (0.09)	0.53 (0.06)	0.64 (0.25)	0	-	-
SWISS-MODEL	[13]	00:41	0.95	0.32 (0.16)	0.37 (0.15)	0.98	0.67 (0.10)	0.66 (0.08)	1	0.84 (0.05)	0.79 (0.04)	0.87 (0.10)	0.69 (0.27)	0.63	0.59 (0.33)	0.71 (0.14)
PRIMO_HHS_3D	[14]	01:09	0.82	0.26 (0.13)	0.33 (0.13)	0.87	0.57 (0.14)	0.57 (0.12)	0.85	0.77 (0.10)	0.72 (0.08)	0.68 (0.09)	0.63 (0.28)	0	-	-
PRIMO_HHS_CL	[14]	01:07	0.86	0.26 (0.13)	0.34 (0.14)	0.89	0.57 (0.15)	0.57 (0.12)	0.89	0.79 (0.09)	0.73 (0.07)	0.68 (0.09)	0.61 (0.28)	0	-	-
PRIMO	[14]	00:52	0.86	0.24 (0.13)	0.33 (0.12)	0.91	0.61 (0.15)	0.60 (0.12)	0.91	0.83 (0.05)	0.77 (0.04)	0.67 (0.09)	0.65 (0.28)	0	-	-
PRIMO_BST_CL	[14]	00:57	0.86	0.24 (0.13)	0.33 (0.12)	0.92	0.61 (0.15)	0.60 (0.12)	0.92	0.83 (0.05)	0.77 (0.04)	0.67 (0.09)	0.65 (0.28)	0	-	-
PRIMO_BST_3D	[14]	00:55	0.80	0.22 (0.12)	0.31 (0.12)	0.85	0.61 (0.15)	0.59 (0.12)	0.85	0.81 (0.06)	0.75 (0.05)	0.67 (0.09)	0.65 (0.28)	0	-	-
Phyre2[b]	[15]	01:30	0.95	0.21 (0.12)	0.31 (0.16)	0.96	0.52 (0.15)	0.60 (0.12)	0.95	0.74 (0.11)	0.75 (0.07)	0.50 (0.02)	0.67 (0.28)	0	-	-
NaiveBLAST[c]	[16]	00:44	0.71	0.19 (0.15)	0.23 (0.14)	0.94	0.59 (0.17)	0.58 (0.14)	0.98	0.81 (0.05)	0.75 (0.05)	0.67 (0.09)	0.65 (0.29)	0	-	-

Notes:
a. The overall sort order is given by the lDDT performance of the hard targets. Values in parenthesis are the standard deviations for the respective scores. The underlying individual target sets may differ and thus cannot result in an absolute performance measure.
b. Some methods do not reflect current developments and are shown in CAMEO for historic comparison.
c. Baseline methods are show for comparison purposes

References

1. Han Y, Zhuang Q, Sun B, et al. Crystal structure of steroid reductase SRD5A reveals conserved steroid reduction mechanism. *Nat Commun*. 2021;12(1):449.
2. Haas J, Gumienny R, Barbato A, et al. Introducing “best single template” models as reference baseline for the Continuous Automated Model Evaluation (CAMEO). *Proteins*. 2019;87(12):1378-1387.
3. Yang J, Anishchenko I, Park H, Peng Z, Ovchinnikov S, Baker D. Improved protein structure prediction using predicted interresidue orientations. *Proc Natl Acad Sci U S A*. 2020;117(3):1496-1503.
4. Song Y, DiMaio F, Wang RY-R, et al. High-resolution comparative modeling with RosettaCM. *Structure*. 2013;21(10):1735-1742.
5. Raman S, Vernon R, Thompson J, et al. Structure prediction for CASP8 with all-atom refinement using Rosetta. *Proteins*. 2009;77 Suppl 9:89-99.
6. Kryshtafovych A, et. al. Prot-00143-2021. *Proteins*. (this issue).

7. Källberg M, Wang H, Wang S, et al. Template-based protein structure modeling using the RaptorX web server. *Nat Protoc*. 2012;7(8):1511-1522.

8. McGuffin LJ, Adiyaman R, Maghrabi AHA, et al. IntFOLD: an integrated web resource for high performance protein structure and function prediction. *Nucleic Acids Res*. 2019;47(W1):W408-W413.

9. McGuffin LJ, Shuid AN, Kempster R, et al. Accurate template-based modeling in CASP12 using the IntFOLD4-TS, ModFOLD6, and ReFOLD methods. *Proteins*. 2018;86 Suppl 1:335-344.

10. McGuffin LJ, Atkins JD, Salehe BR, Shuid AN, Roche DB. IntFOLD: an integrated server for modelling protein structures and functions from amino acid sequences. *Nucleic Acids Res*. 2015;43(W1):W169-W173.

11. Rykunov D, Steinberger E, Madrid-Aliste CJ, Fiser A. Improved scoring function for comparative modeling using the M4T method. *J Struct Funct Genomics*. 2009;10(1):95-99.

12. Yang Y, Faraggi E, Zhao H, Zhou Y. Improving protein fold recognition and template-based modeling by employing probabilistic-based matching between predicted one-dimensional structural properties of query and corresponding native properties of templates. *Bioinformatics*. 2011;27(15):2076-2082.

13. Waterhouse A, Bertoni M, Bienert S, et al. SWISS-MODEL: homology modelling of protein structures and complexes. *Nucleic Acids Res*. 2018;46(W1):W296-W303.

14. Hatherley R, Brown DK, Glenister M, Tastan Bishop Ö. PRIMO: An Interactive Homology Modeling Pipeline. *PLoS One*. 2016;11(11):e0166698.

15. Kelley LA, Mezulis S, Yates CM, Wass MN, Sternberg MJE. The Phyre2 web portal for protein modeling, prediction and analysis. *Nature Protocols*. 2015;10(6):845-858. doi:10.1038/nprot.2015.053

16. Haas J, Barbato A, Behringer D, et al. Continuous Automated Model EvaluatiOn (CAMEO) complementing the critical assessment of structure prediction in CASP12. *Proteins*. 2018;86 Suppl 1:387-398.
