# Supplementary material for: Continuous Automated Model EvaluatiOn (CAMEO)—Perspectives on the future of fully automated evaluation of structure prediction methods
Source: Proteins. 2021 Aug 19;89(12):1977–86. doi: 10.1002/prot.26213 (PMC8673552; doi:10.1002/prot.26213)
Supplement: Supplementary file 2 — Table S2 QE servers comparison for all targets (8594)ᵃ in the 2020 time frame. [file PROT-89-1977-s001.pdf]

Supplementary Table 2: QE servers comparison for all targets (8594)[a] in the 2020 time frame.

Server name	Reference	Response time (hh:mm:ss)	Returned fraction	ROC AUC	PR AUC	Partial ROC AUC	Partial PR AUC
QMEANDisCo 3	1	00:28:25	1	0.94	0.91	0.157	0.137
ModFOLD7_lDDT	2,3	18:17:32	0.82	0.90	0.84	0.134	0.114
ModFOLD6[b]	4	28:36:04	0.55	0.88	0.81	0.125	0.109
ProQ3D_LDDT	5	10:03:22	0.82	0.89	0.82	0.125	0.113
ProQ3	6	10:02:46	0.82	0.87	0.81	0.123	0.104
QMEAN 3[b]	7	00:26:35	1	0.87	0.79	0.119	0.103
ProQ2[b]	8	00:46:45	0.63	0.85	0.77	0.115	0.095
ModFOLD4[b]	9	38:35:00	0.48	0.85	0.77	0.111	0.100
ProQ3D	5	09:51:39	0.82	0.84	0.76	0.109	0.097
Baseline Potential[c]	10	00:26:22	1	0.77	0.69	0.092	0.081
VoroMQA_sw5	11	00:00:16	0.98	0.80	0.69	0.091	0.089
VoroMQA_v2	11	00:00:19	0.98	0.74	0.57	0.052	0.091

Notes:
a. The overall sort order is given by the Partial ROC AUC. The underlying individual target sets may differ and thus cannot result in an absolute performance measure.
b. Some methods do not reflect current developments and are shown in CAMEO for historic comparison.
c. Baseline methods are show for comparison purposes

References

1. Studer G, Rempfer C, Waterhouse AM, Gumienny R, Haas J, Schwede T. QMEANDisCo-distance constraints applied on model quality estimation. *Bioinformatics*. 2020;36(8):2647.

2. Cheng J, Choe M-H, Elofsson A, et al. Estimation of model accuracy in CASP13. *Proteins*. 2019;87(12):1361-1377.

3. Maghrabi AHA, McGuffin LJ. Estimating the Quality of 3D Protein Models Using the ModFOLD7 Server. *Methods Mol Biol*. 2020;2165:69-81.

4. Maghrabi AHA, McGuffin LJ. ModFOLD6: an accurate web server for the global and local quality estimation of 3D protein models. *Nucleic Acids Res*. 2017;45(W1):W416-W421.

5. Uziela K, Hurtado DM, Shu N, Wallner B, Elofsson A. ProQ3D: improved model quality assessments using deep learning. *Bioinformatics*. Published online 2017:btw819. doi:10.1093/bioinformatics/btw819

6. Uziela K, Shu N, Wallner B, Elofsson A. ProQ3: Improved model quality assessments using Rosetta energy terms. *Sci Rep*. 2016;6:33509.

7. Benkert P, Biasini M, Schwede T. Toward the estimation of the absolute quality of individual protein structure models. *Bioinformatics*. 2011;27(3):343-350.

8. Ray A, Lindahl E, Wallner B. Improved model quality assessment using ProQ2. *BMC*

Bioinformatics. 2012;13:224.

9. McGuffin LJ, Buenavista MT, Roche DB. The ModFOLD4 server for the quality assessment of 3D protein models. *Nucleic Acids Res*. 2013;41(Web Server issue):W368-W372.

10. Haas J, Barbato A, Behringer D, et al. Continuous Automated Model EvaluatiOn (CAMEO) complementing the critical assessment of structure prediction in CASP12. *Proteins*. 2018;86 Suppl 1:387-398.

11. Olechnovič K, Venclovas Č. VoroMQA: Assessment of protein structure quality using interatomic contact areas. *Proteins*. 2017;85(6):1131-1145.
